# Synthesis and crystal structure of piperidinyl propanenitrile towards the preparation of piperidine based bioactive films for drug delivery applications

**DOI:** 10.1038/s41598-024-81996-6

**Published:** 2025-01-03

**Authors:** Reham A. Mohamed-Ezzat, Mohamed S. Hasanin, Benson M. Kariuki, Sawsan Dacrory

**Affiliations:** 1 Chemistry of Natural and Microbial Products Department, Pharmaceutical and Drug Industries Research Institute, National Research Centre, Cairo, 12622, Egypt; 2 Cellulose and Paper Department, National Research Centre, Cairo, 12622, Egypt; 3 School of Chemistry, Cardiff University, Main Building, Park Place, Cardiff, CF10 3AT UK

**Keywords:** Piperidine, Synthesis, Crystal structure, Antimicrobial, Sodium alginate, Bioactive films, Materials science, Biomaterials, Soft materials

## Abstract

Compounds containing the piperidine group are highly attractive as building blocks for designing new drugs. Functionalized piperidines are of significant interest due to their prevalence in the pharmaceutical field. Herein, 3-oxo-3-(piperidin-1-yl) propanenitrile has been synthesized, and piperidine-based sodium alginate/poly(vinyl alcohol) films have been prepared. The polymeric films display potency and potential for application to fight against microbial infections. The films could also help maintain interaction with tissue to ensure the controlled release of therapeutic molecules. Thus, they are promising in developing drug delivery systems essential in the pharmaceutical industry. The structure of the 3-oxo-3-(piperidin-1-yl)propanenitrile was confirmed via spectroscopic and single crystal x-ray diffraction techniques. A homogenous solution of sodium alginate (SA) was used to prepare the film by the casting method in the presence of poly(vinyl alcohol) (PVA) and 3-oxo-3-(piperidin-1-yl)propanenitrile (PPN). The prepared films were characterized physiochemically via FTIR, XRD, and TGA. The film morphology was studied using SEM. The antimicrobial potency of the prepared films was assessed against various species of microorganisms. The physicochemical analysis indicated that the films were bound by chemical and physical bond formation between the cyano group of 3-oxo-3-(piperidin-1-yl)propanenitrile, methylene group of PVA, and the hydroxyl group of SA. The films showed smooth, homogenous surfaces and good mechanical properties. The results revealed that the films are bioactive, as indicated by promising antimicrobial potency against *P. aeruginosa*,* S. aureus*,* E. coli*,* B. subtilis*, and *C. albicans*, with high potency as well as moderate activity against *A. niger*. Polymeric films have promising potential to be utilized in drug delivery applications.

**Supplementary Information:**

The online version contains supplementary material available at 10.1038/s41598-024-81996-6.

## Introduction

Piperidines are prevalent compounds and are amongst the most common heterocycles in medications approved by the Food and Drug Administration (FDA). They are also widely present in drug candidates^1^. Thus, robust methods for synthesizing these heterocycles are desirable for the efficient probing of structure-activity relationships during, for example, drug discovery investigation for pharmaceutical applications^2–4^. Moreover, developments in synthetic approaches toward new piperidines are necessary for discovering novel medicines to add to the existing strategies^5–10^.

Piperidine-containing compounds are also attractive as building blocks for new pharmaceuticals for treating various conditions^11–13^. Functionalized piperidines and the synthetic techniques utilized to produce them are of substantial current importance in pharmaceutical research^14^. The piperidine ring is a core structure of several alkaloid natural products^15^, as well as many synthetic pharmaceuticals such as aminoglutethimide, trihexyphenidyl, minoxidil, n-butyl-deoxynojirimycin (Miglustat), lenalidomide, argatroban, paroxetine (Paxil), clopidogrel (Plavix), tadalafil (Cialis), cyproheptadine, loratadine and mefloquine(Lariarn)^16^.

Many piperidine derivatives are endowed with antimicrobial potencies. Some analogs isolated from *Streptomyces ficellus*, exemplified by nojirimycin, (-)-swainsonine, quinine, (S)-conline, and (S)-anabasine are effective against *E. coli*, *S. lutea*, and *S. aureus.* Nojirimycin is considered prototypical of a novel category of antibiotics. Additionally, many other piperidine derivatives exhibit potent activity against various fungal strains, including *Aspergillus flavus*, *Candida-51*, *Candida 6*, *A. flavus*, *A. niger*, and *C. neoformans*^17^.

Many delivery systems have been designed in recent years to address the pharmacokinetic drawbacks associated with poor permeability, low water solubility, and undesirable distribution, metabolism, and excretion pathways of many active pharmaceutical ingredients^18,19^. It is widely acknowledged that polymeric materials have the necessary physicochemical and biological properties to facilitate the administration of bioactive molecules^20^, enabling the achievement of appropriate concentration at the right site for a sufficient duration to produce the desired response^21,22^. Consequently, there has been a lot of interest in developing polymeric formulations and delivery systems with different architectures, such as nanoparticles, dendrimers, films, and gels^23^, hydrogels^24^, and prodrugs to modulate the amount, and rate of drug release^25,26^. Alginates have attracted much attention in biomaterial research for many applications, including scaffolds for tissue engineering, drug delivery, and cell encapsulation^27^.

The drug delivery system is part of the strategy utilized widely in the pharmaceutical industry to improve the transport of drugs to targets as well to control the release^28^. As drug delivery systems, polymeric films can maintain contact with the target tissue and enable the controlled release of bioactive molecules^29^. Recently, we have synthesized polymeric conjugates based on heterocyclic compounds^30,31^ and several pharmaceutical agents based on heterocycles with the potential to impact medicinal chemistry^31^. Polysaccharides have played a key role in the formulation of drugs and the setup of drug delivery systems^32,33^. These types of biopolymers could improve the solubility properties, regulate the sensitivity of active ingredients to pH and temperature, and enhance storage stability. SA is a linear polysaccharide derivative of alginic acid comprising α-l-guluronic and 1,4-β-d-mannuronic acids obtained from marine brown algae cell walls^34^. SA is a water-soluble anionic polymer that could prove superior to synthetic polymers if better understood and performance optimized. SA-based systems for cancer-targeted medication delivery need to be updated and built more methodically because alginate and its derivatives have found new uses in recent years with high biocompatibility and safety profiles^35^. In this context, PVA is a water-soluble hydrophilic polymer^36^. PVA is a popular option among water-soluble polymers for gel formation because it has several beneficial properties, including high water content, ease of machinability, low toxicity, and good strength^37^. PVA has been used in several medical, pharmaceutical, agriculture, and industrial applications^38^.

Consequently, drug carrier SA and PVA formulations could be synergetic for both polymers. Therefore, this study explores a novel conjugate based on SA and PVA containing the piperidine group with different concentrations. Physicochemical and morphology analyses were carried out to confirm the structure of the prepared films. The formulated films’ mechanical properties and antimicrobial activities were also evaluated.

## Experimental

### Materials

Fisher Scientific UK provided sodium alginate (SA), and polyvinyl alcohol (PVA). All chemicals, microbial media, and reagents used were of pure analytical grade.

### **Synthesis of 3-oxo-3-(piperidin-1-yl)propanenitrile (3**)

Piperidine (**2**) (0.02 mol) was added to ethylcyanoacetate (**1**) (0.01 mol) in the presence of ethanol (20 ml), and the reaction mixture was allowed to stir at room temperature for two h. The reaction mixture was then filtered to give a white precipitate, which was crystallized from ethanol to afford compound (**3**) as colorless crystals, mp.78-79^o^C, 92% yield; ^1^H NMR (500 MHz, DMSO-*d*_*6*_): δ 1.39–1.53 (*m*, 6 H, piperidine), 3.24–3.37 (*m*, 4 H, piperidine), 3.97 (*s*, 2 H, CH_2_). Anal. Calcd. For. C_8_H_12_N_2_O (152.19): C, 63.13; H, 7.95; N, 18.41. Found: C, 63.10; H, 7.91; N, 18.38.

### Crystal structure determination

Single crystal XRD data were collected using an Agilent SuperNova Dual Atlas diffractometer at room temperature with a mirror monochromator utilizing Mo (λ = 0.7107 Å) radiation. The crystal structure was solved using SHELXT^40^ and refined using SHELXL^41^. Non-hydrogen atoms were refined with anisotropic displacement parameters. Hydrogen atoms were inserted in idealized positions, and a riding model was used with Uiso set at 1.2 or 1.5 times the value of Ueq for the atom to which they are bonded. C_8_H_12_N_2_O, Monoclinic, P2_1_/c, FW = 152.20, T = 293(2) K, λ = 0.71073 Å, a = 9.7133(7) Å, b = 8.9517(5) Å, c = 9.8332(8) Å, α = 90°, β = 101.427(7)°, γ = 90°, V = 838.05(10) Å^3^, Z = 4, ρ = 1.206 Mg/m^3^, µ = 0.082 mm^-1^, crystal size = 0.570 × 0.360 × 0.190 mm^3^, reflections collected = 7202, independent reflections = 2069, R(int) = 0.0352, goodness-of-fit on F^2^ = 1.076, R1(I > 2σ(I)) = 0.0498, wR2(I > 2σ(I)) = 0.1273, R1 (all data) = 0.0726, wR2 (all data) = 0.1443, extinction coefficient = 0.119(9). The crystal structure has been deposited in the CSD under reference number CSD 2304617.

### Preparation of the bioactive film

A homogenous solution of SA was prepared by dissolving 5 g of SA in 100 mL distilled water with continuous stirring to complete dissolution. 10 mL of the SA solution was then added to 10 mL of PVA (5%). The mixture was stirred for 10 min to achieve homogeneity. 3-Oxo-3-(piperidin-1-yl)propanenitrile (PPN) with different concentrations, namely (0.05, 0.1, 0.15 g), was then added individually with continuous stirring to prevent agglomeration. The precursor was finally poured into a Teflon dish and dried at room temperature.

#### Characterization

FT-IR spectra were obtained on a Shimadzu 8400 S FT-IR Spectrophotometer in the range of 400–4000 cm^− 1^. The ^1^H NMR spectrum was recorded on Bruker Fourier at 500 MHz at 300 K. The analysis of the surface morphology was carried out utilizing a scanning electron microscope (SEM), a JEOL JEM-2100 electron microscope at 100 kV magnification, and an acceleration voltage of 120 kV without coating. The powder XRD patterns were recorded on a Diano X-ray diffractometer or a Philips X-ray diffractometer (PW 1930 generator, PW 1820 goniometer) with CuK radiation sources (λ = 0.15418 nm), at diffraction angles in the 2θ range 10 to 80° in reflection mode. Mechanical properties of the prepared films were estimated utilizing a Lloyd LR instrument (England) under elongation at a constant temperature environment with a speed of 5 mm/minute and a fixed gauge of 20 mm. Thermogravimetric analysis (TGA) was performed utilizing the TGA Q500 device with a heating rate (10ºC/min). The temperature ranged from room temperature up to 700 ºC under air atmosphere.

### Microbiological evaluation

The antimicrobial activity of the prepared films was assessed using the turbidimetric method described in our previous work^39^ against popular foodborne microorganisms, including the Gram-positive bacterial strains *Bacillus subtilis* ATCC 6051and *Staphylococcus aureus* ATCC 25,923, and the Gram-negative *Escherichia coli* ATCC25922 and *Pseudomonas aeruginosa* ATCC 27,853. Fungal strains were unicellular and filamentous fungus strains, namely *Candida albicans ATCC90028* and *Aspergillus niger* RCMB 02724, respectively. The selected microorganisms were incubated in a nutrient broth medium for 24 h at 37 °C. The fungal strains were grown on potato dextrose broth medium (PDB) plates and incubated at 30 °C for 3–5 days. Streptomycin and Griseofulvin were the standard broad-spectrum antibacterial and antifungal, respectively.

## Result and discussion

### Synthesis of 3-oxo-3-(piperidin-1-yl)propanenitrile (PPN) (3)

**Fig. 1:**
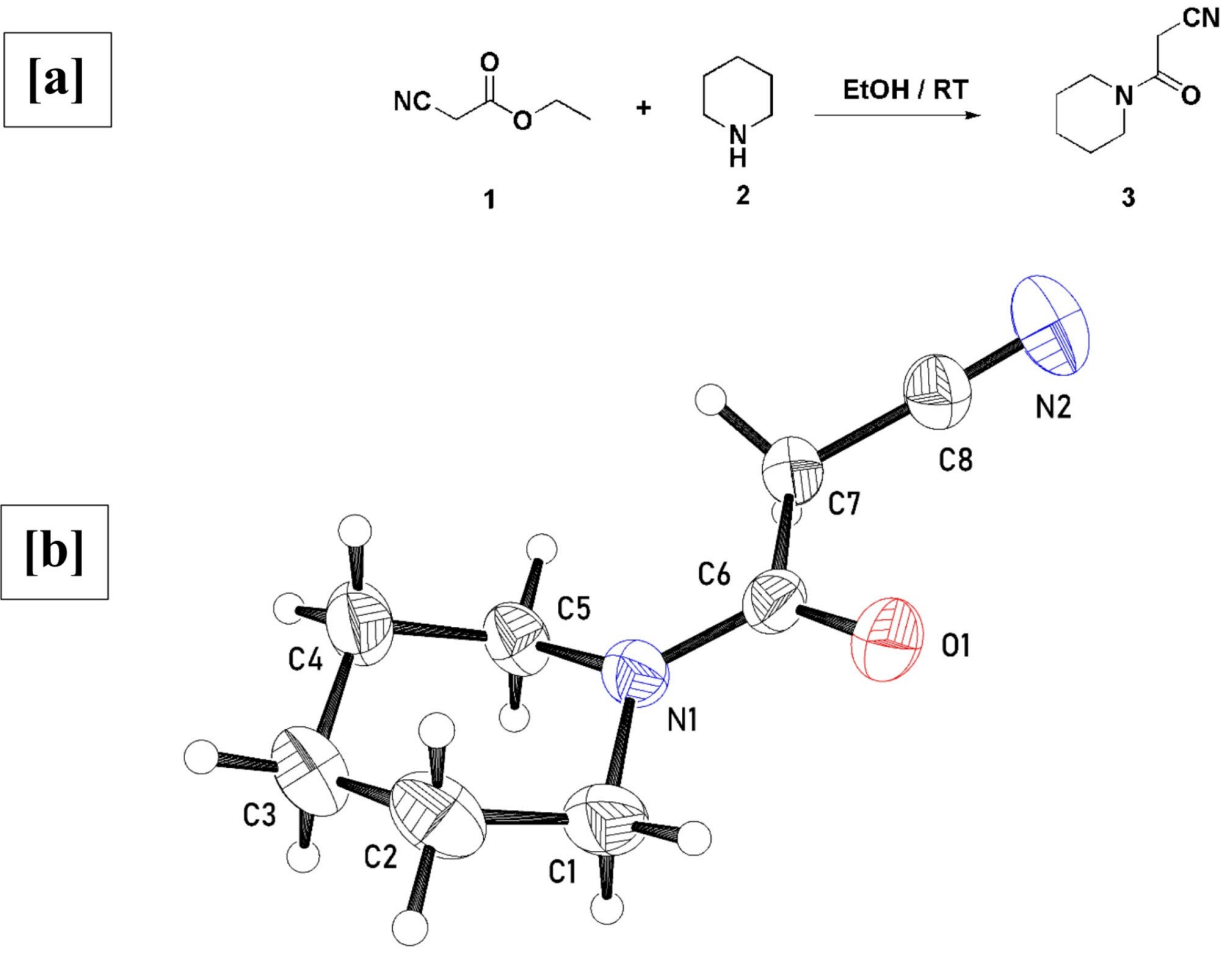
(**a**) PPN synthesis and (**b**) An Ortep representation of the molecule of compound **3** (PPN).

Compound **3** was obtained by reacting ethylcyanoacetate and piperidine in ethanol with stirring for two h (Fig. 1a). The chemical structure of **3 (PPN)** was assigned based on its spectral data. The ^1^H NMR spectrum of the 3-oxo-3-(piperidin-1-yl)propanenitrile (PPN) (3) (Figure S1) reveals multiplet signals at δ 1.39–1.53 & 3.24–3.37 ppm assignable to the methylene protons of piperidine, as well as the signal of the CH_2_ group at δ 3.97 ppm.

### Crystal structure

Crystal structure determination confirmed the identity of compound **3** (Fig. 1b). The crystal structure obtained was the same as that previously reported^44^. In the molecule, the piperidine ring (C1-C5, N1) is in chair conformation and the 2-cyano-*N*,*N*-dimethylacetamide fragment (C6-C8, N2, O1) is planar.

### Preparation of films

**Fig. 2:**
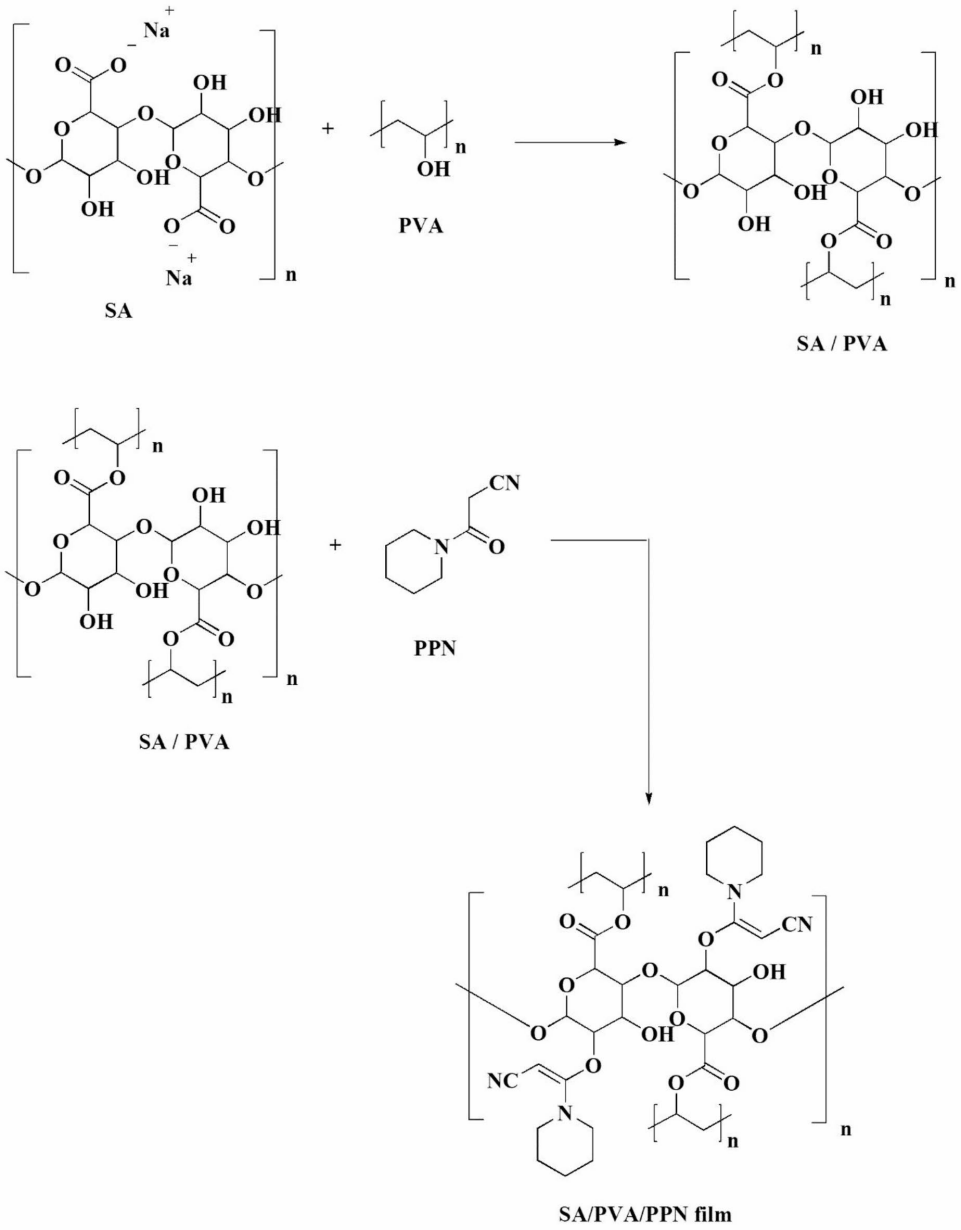
Suggested mechanism of film formulation.

The films were prepared by chemical and physical bond formation between the 3-oxo-3-(piperidin-1-yl)propanenitrile, the carboxylic groups of PVA, and the hydroxyl groups of SA as proposed and illustrated in Fig. 2. The interaction occurred via a two-step approach: (i) Crosslinking of PVA with SA. (ii) Condensation reaction of the PVA/SA with the 3-oxo-3-(piperidin-1-yl)propanenitrile (PPN). Other physical interaction may have occurred, as PPN can be interacted with the SA chain due to its being well-dispersed by the casting method^42^. The photographs of the SA/PVA/PPN with different concentrations of PPN were presented in Figure S2 show transparency with a brownish appearance. The brownish colour increased with the PPN content showing high concentration (0.15) of PPN as less transparent with high opacity.

### FTIR analysis

**Fig. 3:**
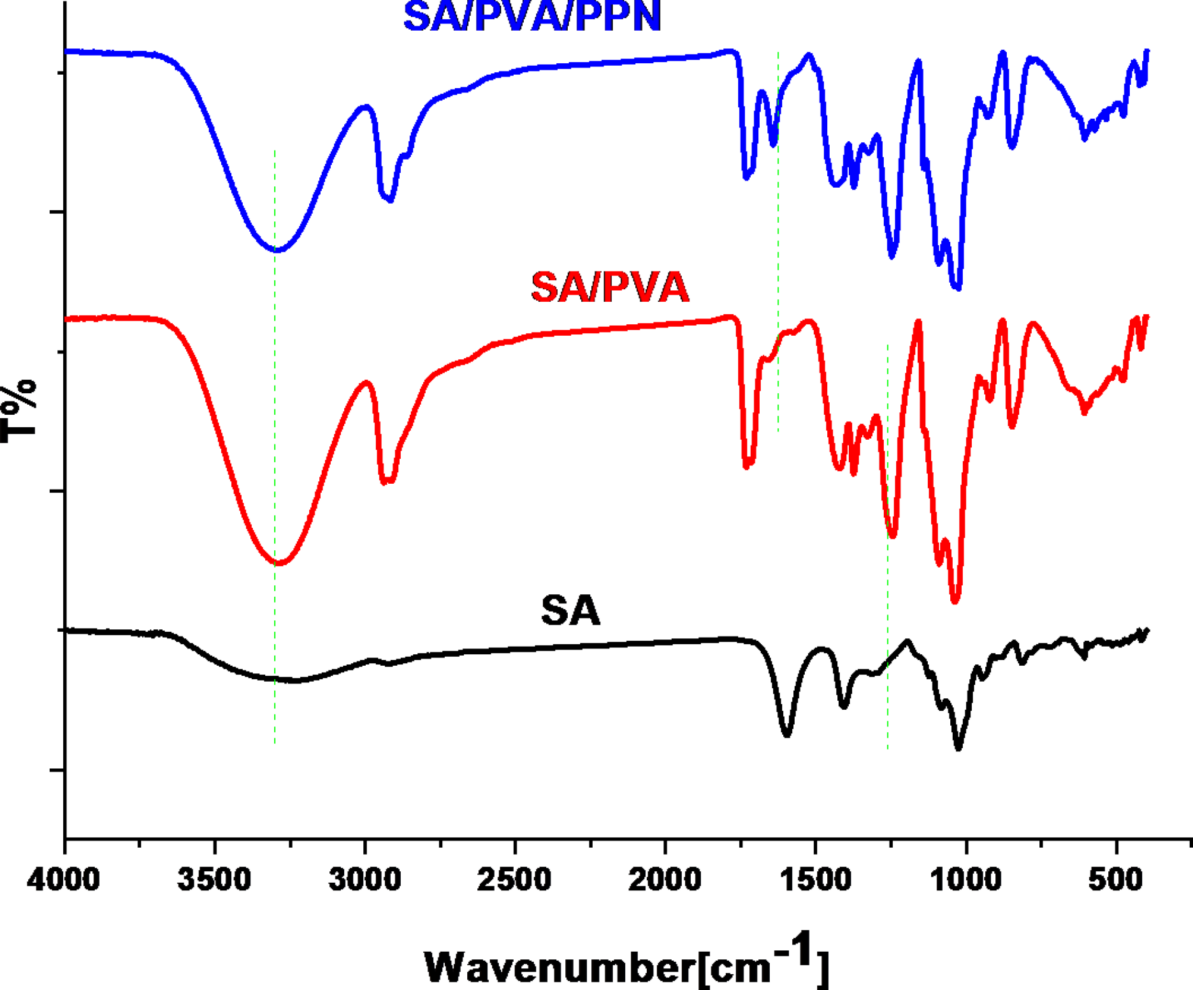
FTIR spectra of SA, SA/PVA, and SA/PVA/PPN films.

Figure 3 shows FTIR spectra of SA, SA/PVA, and SA/PVA/PPN film (0.15 g). SA shows broad bands at 3500 cm^− 1^, 1600 cm^− 1^, 1400 cm^− 1,^ and 1000 cm^− 1^ corresponding to OH stretching vibration, symmetric and asymmetric COO − stretching vibrations, and C-O-C stretching vibration of the ether linkage, respectively^43^. The SA/PVA spectrum displays sharp bands at 3500 cm^− 1^, 2900 cm^− 1^, 1720 cm^− 1^, 1200 cm^− 1^, 1030 cm^− 1^, and 850 cm^− 1^ attributed to OH, C-H, carboxylic group C = O, and O-CH_2_ stretching, and C-O ether linkage and C = C bending, respectively^44^. In addition, the cyano group (CN) found in the crystal structure of PPN disappeared completely in the prepared film of SA/PVA/PPN. In contrast, the C = N vibration appeared clearly at 1629 cm^− 1^ due to the new amide group formation. The band at 2846 cm^− 1^ is possibly due to the incorporation of PPN into SA/PVA, and the band at 1720 cm^− 1^ decreased in intensity, whereas the band at 1650 cm^− 1^ remained. In summary, the incorporation of PPN into SA/PVA is evident in the FTIR spectrum, as shown by a significant change in the film functional groups^45^.

### Scanning electron microscopy (SEM)

**Fig. 4:**
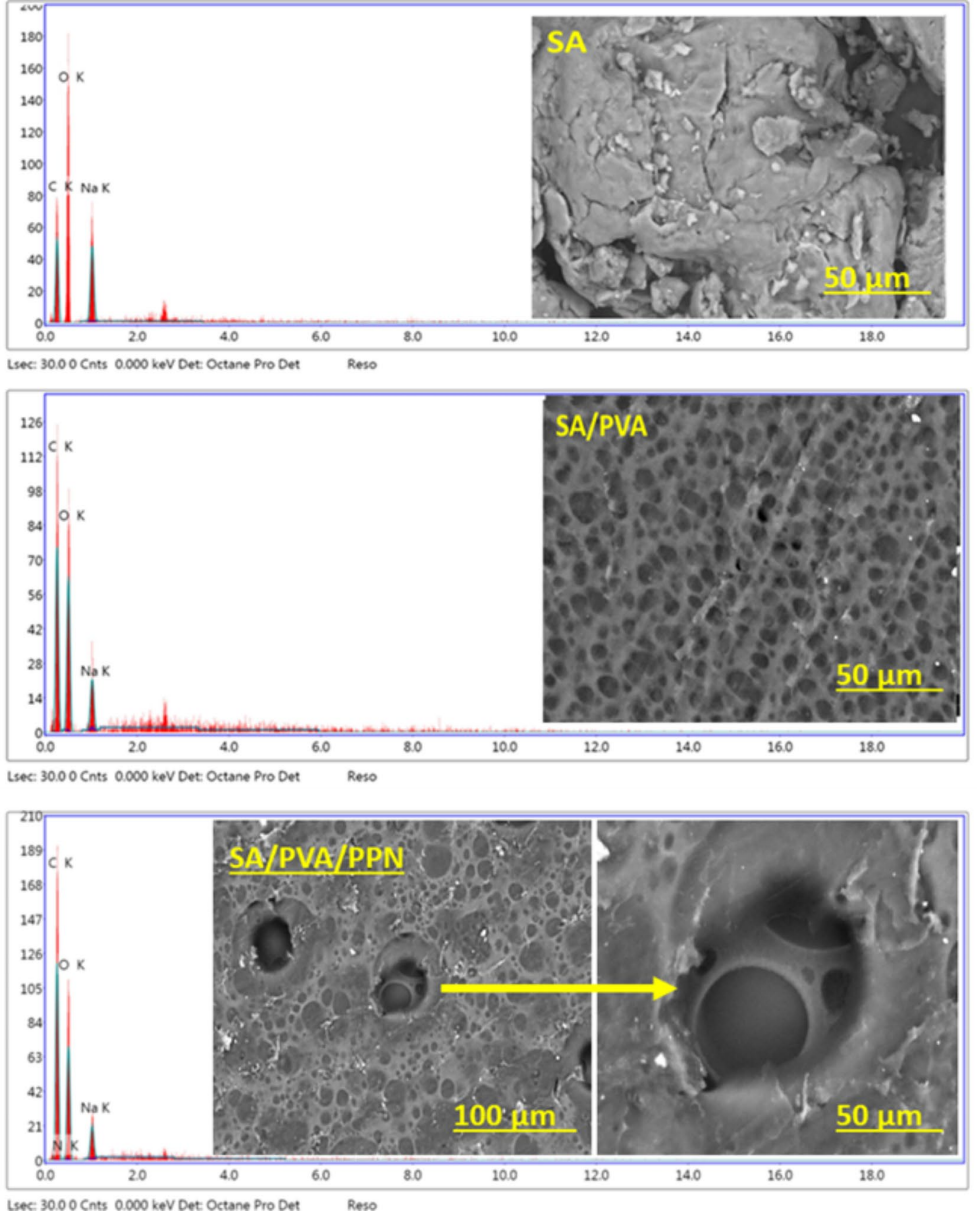
SEM and EDX of SA, SA/PVA and SA/PVA/PPN films.

SEM has been utilized to study the surface morphology of SA, SA/PVA, and SA/PVA/PPN (0.15 g) films. Figure 4 illustrates the surface of SA, which has a compact, rough texture. The EDX chart of SA shows the presence of carbon, oxygen, and sodium, typical elemental content of pure SA. The SEM image of SA/PVA shows a surface that has become porous due to the interaction of SA and PVA. The EDX chart of SA/PVA shows a change in the element percentage compared to pure SA due to the incorporation of PVA. However, the SEM image of the SA/PVA/PPN film showed decreased pores, possibly due to strong physical interaction between PPN and the SA chain. The EDX chart of SA/PVA/PPN film is the same as that of SA/PVA with additional nitrogen and a change in the elemental ratio, indicating that PPN was trapped in SA/PVA pores. The results of the topographical study are consistent with the formulation of PPN into the SA/PVA system.

### Powder X-ray diffraction

**Fig. 5:**
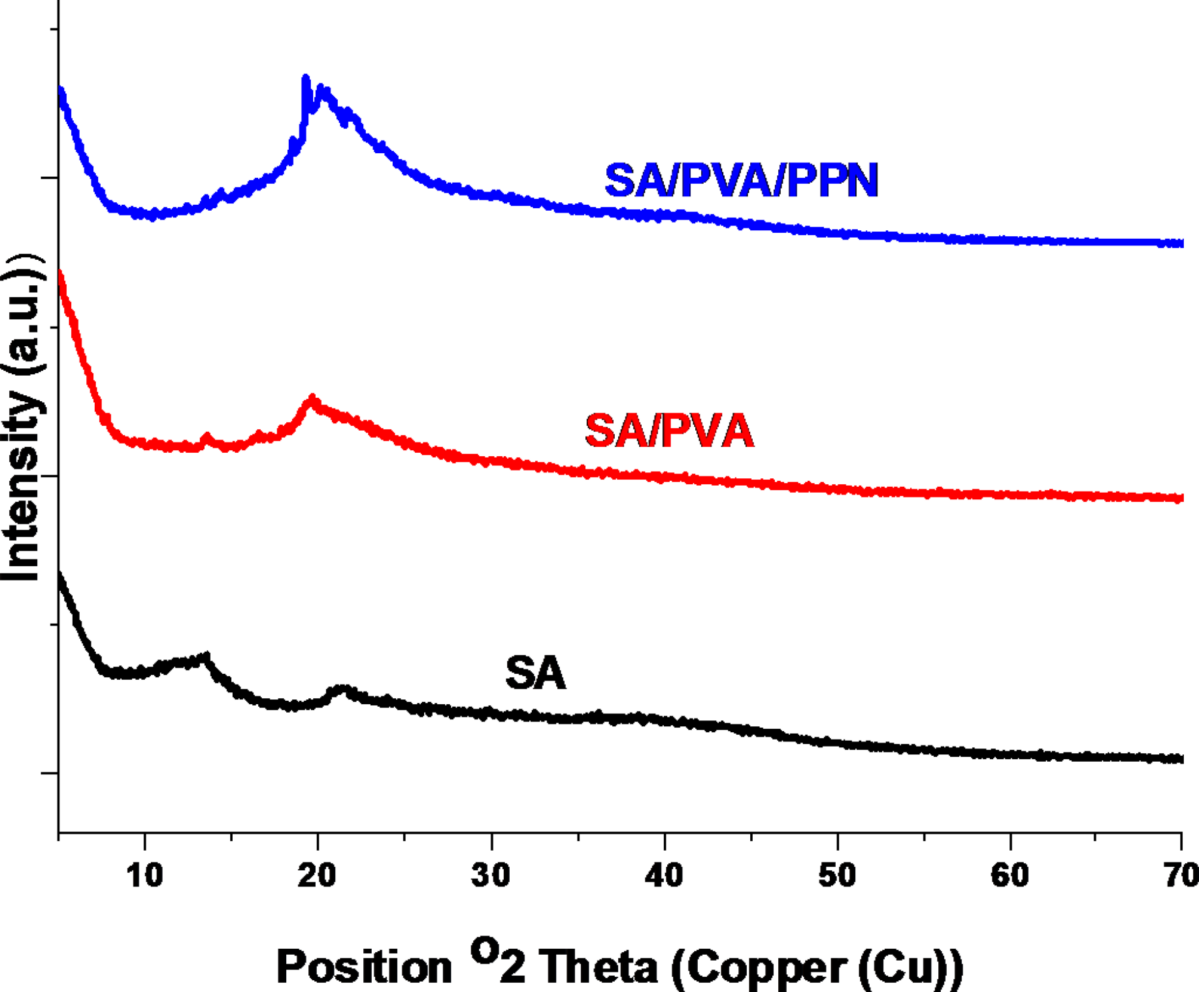
XRD of SA, SA/PVA and SA/PVA/PPN films.

XRD patterns of SA, SA/PVA and SA/PVA/PPN (0.15 g) are presented in Fig. 5. SA pattern has two peaks at 2θ, approximately at 13° and 21°, which are typical of the XRD of pure SA with a semi-crystalline nature, according to the literature^46,47^. In this context, incorporating PVA into SA affects the SA XRD pattern where the intensity of the low-angle peak increases and the high-angle peak intensity decreases. Moreover, the incorporation of PPN significantly affected the XRD pattern, which appeared as a single broad peak at 2θ around 21^o^ with the diminution of low-angle peaks. The broad peaks (for example, seen for pure SA at 2θ approximately at 13° and 21^o^) indicate poor sample crystallinity. This is not unexpected for a polymeric material. In the case of SA/PVA, crystallinity is marginally higher, as shown by the sharper peak (though weaker) peak at 13°. The crystallinity of SA/PVA/PPN remains higher as the sharper peak at 19 ^o^ shows.

### Mechanical properties

**Fig. 6:**
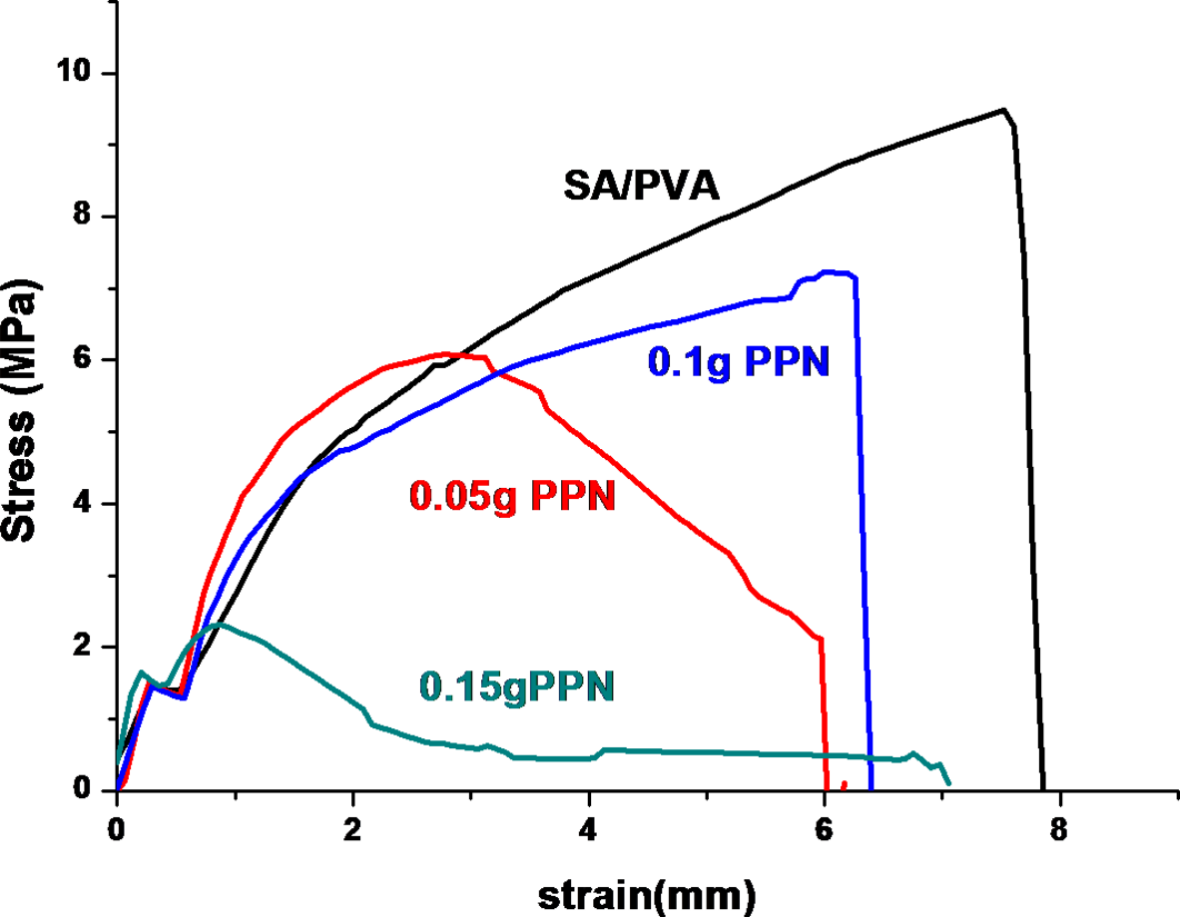
Mechanical properties of SA/PVA and SA/PVA/PPN films.

The mechanical strength of the films was assessed by a study of their elongation ability, as presented in Fig. 6. The data shows that SA/PVA has good mechanical properties with stress and strain of 8.5 MPa and 8 mm, respectively. PNN decreases the stress and strain of SA/PVA film, with the effect of 0.15 g PNN being more significant than that of 0.1 g and 0.05 g PPN. These observations show that trapping PPN particles into the polymer network and the accompanying attraction of the function groups that decrease the interaction force between polymer chains leads to a decrease in the strain and stress loading.

### Thermogravimetric analysis

**Fig. 7:**
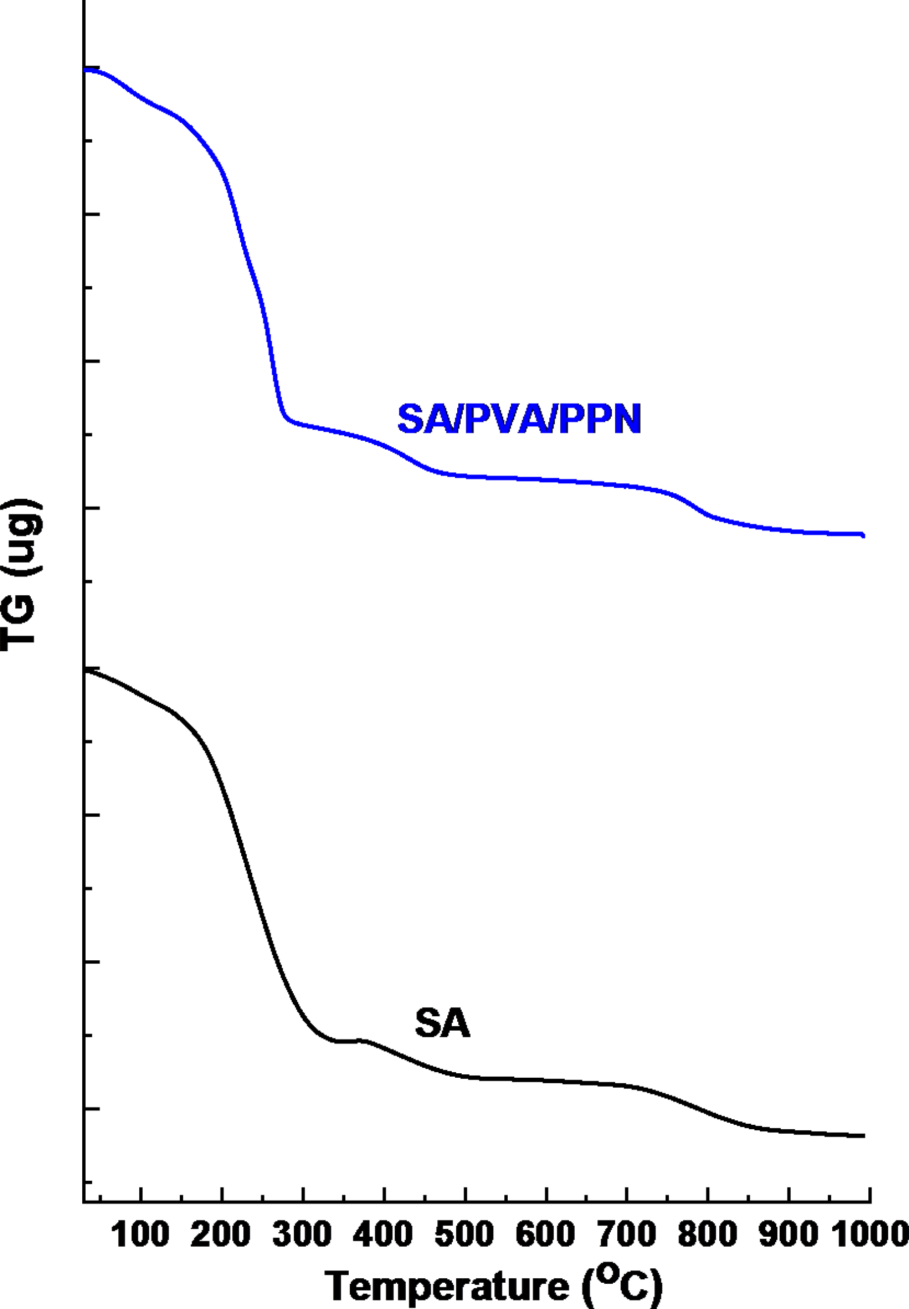
Thermogravimetric analysis of neat SA and SA/PVA/PPN.

Figure 7. shows the thermal analysis of the neat SA and SA/PVA/PPN (0.15 g). The degradation of SA occurred in three main stages. The first step up to 150 C represents moisture removal; the second step occurred between 150 and 289 °C and is attributed to decomposition through the pyrolysis process, with the weight falling to about 31%. The third step was between 330 and 482 °C, representing the conversion of organic material to carbon with weight falling to about 16%. In this context, the weight remaining after thermal degradation was recorded as about 9%, and these above findings, according to reports from other researchers, are consistent with pure SA^48–50^. SA/PVA/PPN degradation also occurred in four steps. The first was moisture removal to 150^o^C, then the second step started at a temperature between 150 and 268 °C, with the weight falling to about 33%. The third step is between 290 and 451 °C, the weight falling to about 20%. The fourth step was between 505 and 825 °C, with 7% as the final weight remaining as well.

### Antimicrobial study

**Fig. 8:**
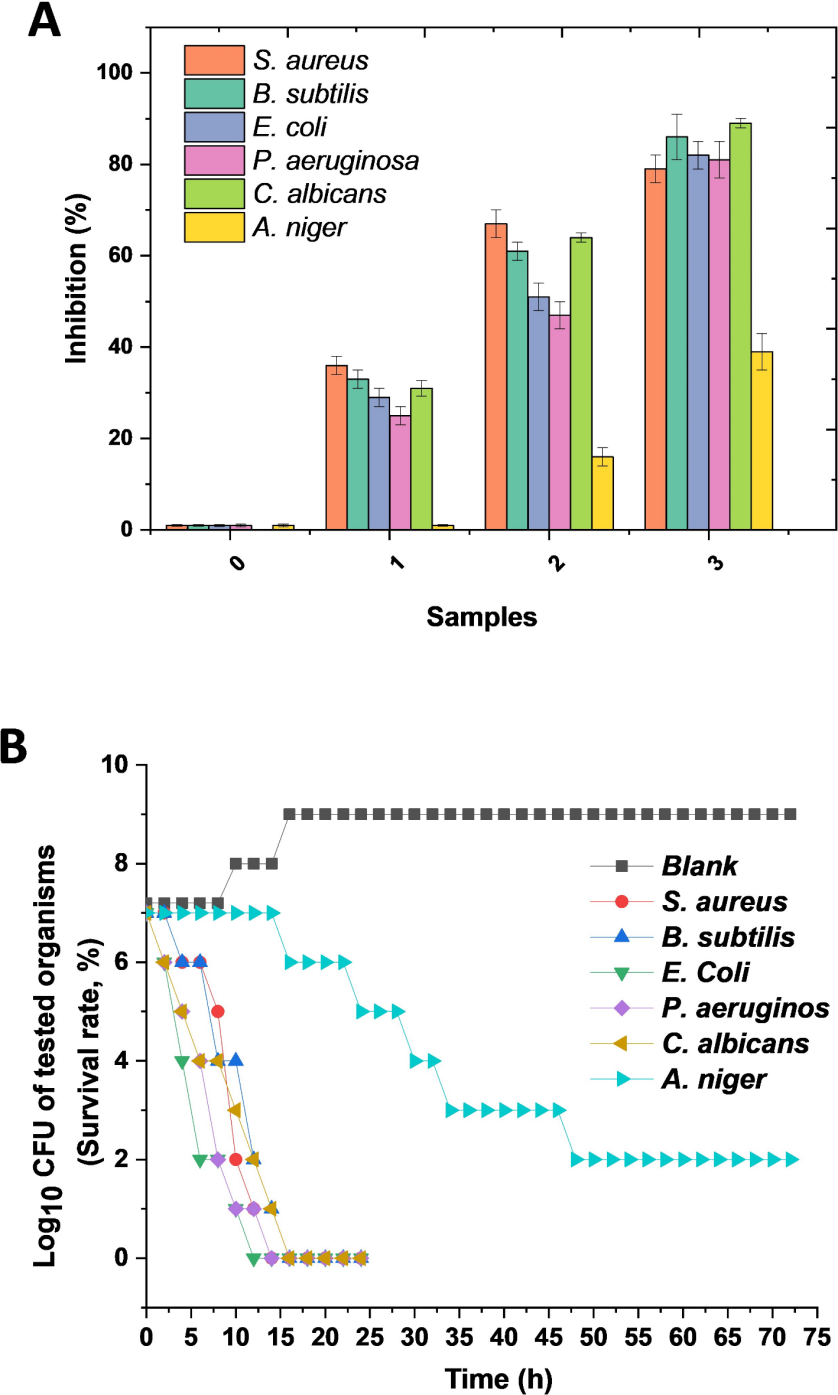
Antimicrobial study of prepared films, antimicrobial activity via turbidimetric assay for (SA/PVA (0), SA/PVA/PPN 0.05 (1), 0.1 (2), and 0.15 g (3)) (**A**) and time required for killing of concentration 0.15 g PNN (**B**).

The antimicrobial study was conducted against six microorganisms, including bacterial strains (Gram-negative and Gram-positive) and fungi strains (unicellular and filamentous), and the time required for killing is presented in Fig. 8. The antimicrobial activity using the turbidimetric method presented in Fig. 8A indicates that good antimicrobial activity was observed, increasing in line with PPN content. Clearly, sample 0 (without PNN) showed no antimicrobial activity in comparison with the other samples (1–3), which PPN (0.05–0.15 g). The antimicrobial activity in samples 1–3 is ascribed to the different PPN concentrations. Sample 3 performed best with inhibition percentages above 80% for all bacterial strains and unicellular fungi. However, the activity against filamentous fungi was about 40%. These observations imply that the PPN plays a role in antimicrobial activity and performs as a strong broad antibacterial and anticandidal with moderate anti-filamentous fungi effect. This may be due to the strong structure of the filamentous fundal at the cellular structure level compared with bacterial and unicellular fungi^51–53^. The rate of killing by sample 3 against the tested microorganisms is presented in Fig. 8B. For the Gram-positive bacteria, the effect started after four h and complete death after 14 h. The Gram-negative bacteria showed an effect after two h, with complete cell death recorded after 12 h. For the unicellular fungi, the behavior was close to that for the bacteria. The filamentous fungi showed no effect for the first eight h, followed by slight growth after 9 h, and no inhibition was observed in 72 h^54^. The time rates of action agreed with traditional antimicrobial studies and affirmed the strong broad-spectrum antibacterial activity of the bioactive film 3 and anticandidal activity. In addition, the test on filamentous fungi indicated deactivation of fungal growth without killing the cells, which could be described as *f*ungistatic^55^.

## Conclusion

3-Oxo-3-(piperidin-1-yl)propanenitrile was synthesized and confirmed using X-ray diffraction and spectroscopic techniques. The piperidine-based sodium alginate/PVA films were then prepared and investigated via FTIR, SEM, and powder XRD. The PPN affects the crystallinity of the polymer network structure and enhances the thermal stability as well. The films showed smooth, homogenous surfaces and good mechanical properties. The results revealed that the films were bioactive, exhibiting promising antimicrobial activities. In this context, the time required for inhibition rate by the active films with the highest ratio of PPN was greatest for Gram-positive and Gram-negative bacteria as well as for unicellular fungi. However, the filamentous fungi show a moderate effect that could be described as *f*ungistatic. The results show promise for using polymeric film for drug delivery applications.

## Electronic supplementary material

Below is the link to the electronic supplementary material.

Supplementary Material 1

## Data Availability

The datasets generated during and/or analyzed during the current study are available from the corresponding author upon reasonable request.
