# Supplementary material for: Synthesis and crystal structure of piperidinyl propanenitrile towards the preparation of piperidine based bioactive films for drug delivery applications
Source: Sci Rep. 2025 Jan 3;15:705. doi: 10.1038/s41598-024-81996-6 (PMC11698915; doi:10.1038/s41598-024-81996-6)
Supplement: Supplementary file 1 — Supplementary Material 1 [file 41598_2024_81996_MOESM1_ESM.docx]

**Synthesis and crystal structure of piperidinyl propanenitrile towards the preparation of piperidine based bioactive films for drug delivery applications**

Reham A. Mohamed-Ezzat^1^, Mohamed S. Hasanin^2*^, Benson M. Kariuki^3^, Sawsan Dacrory^2^

^1^Chemistry of Natural and Microbial Products Department, Pharmaceutical and Drug Industries Research Institute, National Research Centre, Cairo, Egypt.

^2^Cellulose and Paper Department, National Research Centre, Cairo, Egypt,

^3^School of Chemistry, Cardiff University, Main Building, Park Place, Cardiff CF10 3AT, UK.

Corresponding author: Mohamed S. Hasanin, email: [sido_sci@yahoo.com](mailto:sido_sci@yahoo.com)


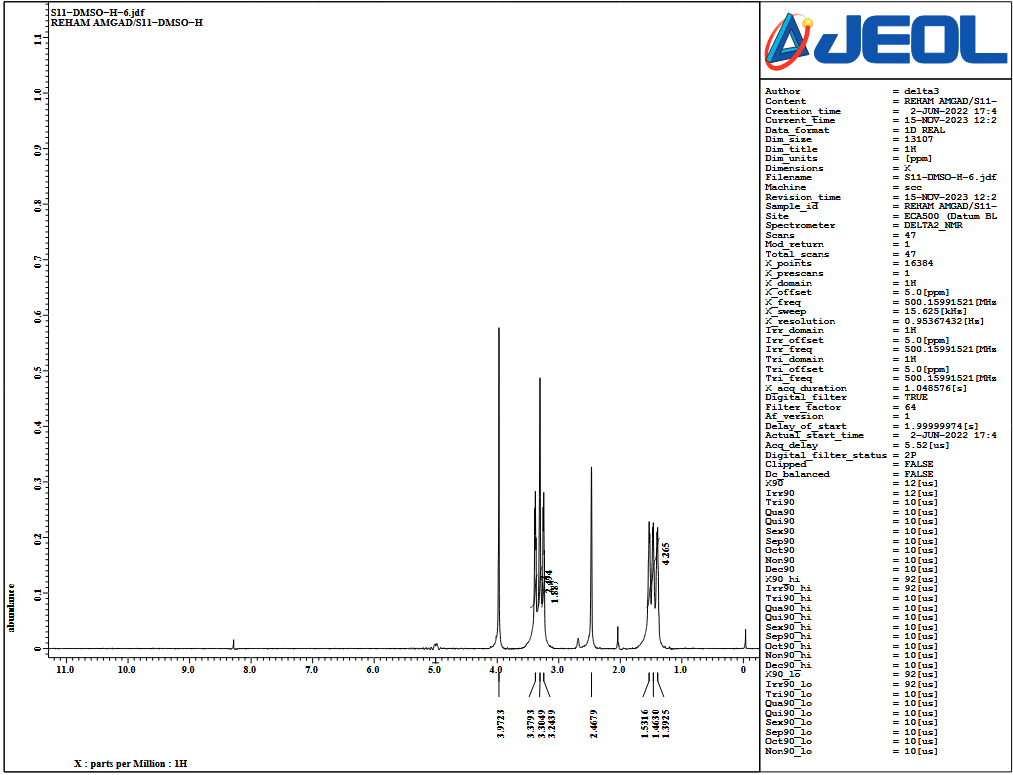


**Figure S1:** 1H-NMR spectrum of compound 3.

| 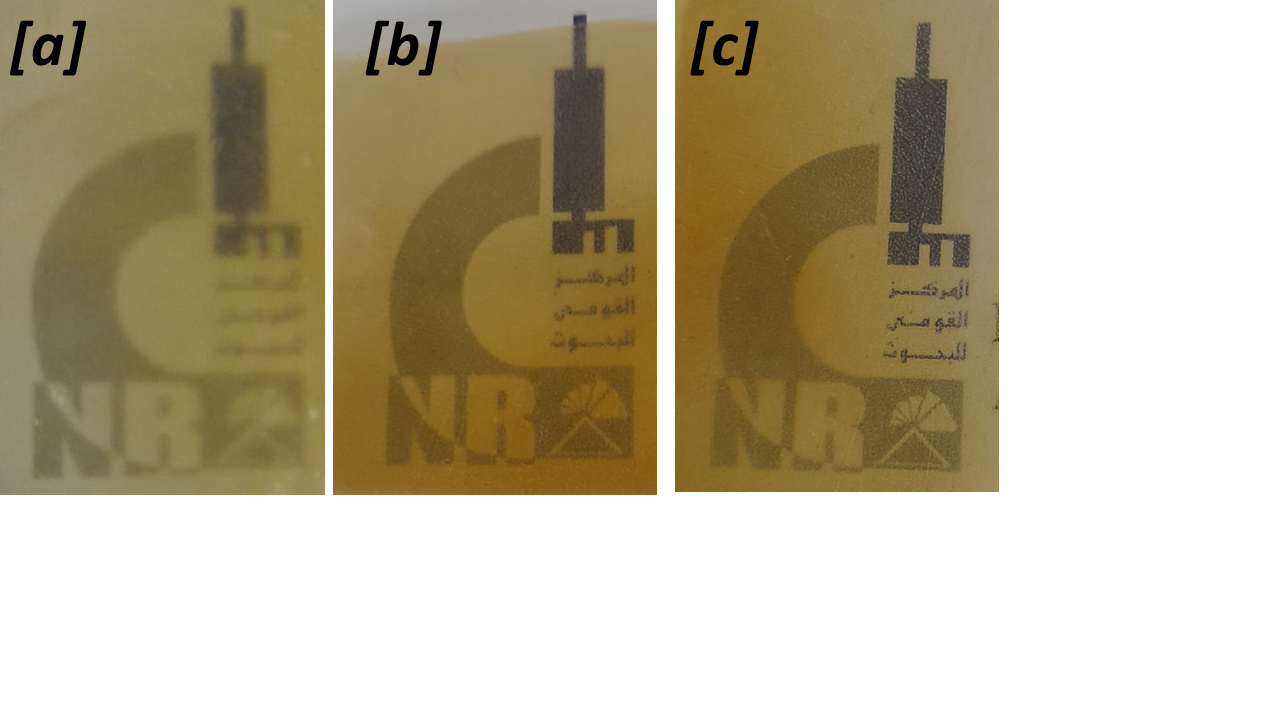 |
| --- |
| **Figure S_2_.** Photography photos of SA/PVA/PPN films with concentrations 0.05 (a), 0.1 (b), 0.15g (c). |
